# Treatment of Acute Mesenteric Ischemia: Individual Challenges for Interventional Radiologists and Abdominal Surgeons

**DOI:** 10.3390/jpm13010055

**Published:** 2022-12-27

**Authors:** Arne Estler, Eva Estler, You-Shan Feng, Ferdinand Seith, Maximilian Wießmeier, Rami Archid, Konstantin Nikolaou, Gerd Grözinger, Christoph Artzner

**Affiliations:** 1 Diagnostic and Interventional Radiology, University Hospital Tuebingen, 72076 Tübingen, Germany; 2 Faculty of Medicine, University of Tuebingen, 72074 Tübingen, Germany; 3 Institute for Clinical Epidemiology and Applied Biometrics, Medical University of Tübingen, 72076 Tübingen, Germany; 4 Department of General & Transplant Surgery, University Hospital Tuebingen, 72076 Tübingen, Germany

**Keywords:** acute mesenteric ischemia, revascularization, laparoscopy

## Abstract

Background: Acute mesenteric ischemia (AMI) is a life-threatening condition resulting from occlusion of the mesenteric arterial vessels. AMI requires immediate treatment with revascularization of the occluded vessels. Purpose: to evaluate the technical success, clinical outcomes and survival of patients receiving endovascular treatment for AMI followed by surgery. Material and Methods: A search of our institution’s database for AMI revealed 149 potential patients between 08/2016 and 08/2021, of which 91 were excluded due to incomplete clinical data, insufficient imaging or missing follow-up laparoscopy. The final cohort included 58 consecutive patients [(median age 73.5 years [range: 43–96 years], 55% female), median BMI 26.2 kg/m^2^ (range:16.0–39.2 kg/m^2^)]. Periinterventional imaging regarding the cause of AMI (acute-embolic or acute-on-chronic) was evaluated by two radiologists in consensus. The extent of AMI and the degree of technical success was graded according to a modified TICI (Thrombolysis in Cerebral Infarction scale) score (TICI-AMI) classification (0: no perfusion; 1: minimal; 2a < 50% filling; 2b > 50%; 2c: near complete or slow; 3: complete). Lab data and clinical data were collected, including the results of follow-up laparoscopy. Non-parametric statistics were used. Results: All interventions were considered technically successful. The most common causes of AMI were emboli (51.7%) and acute-on-chronic thrombotic occlusions (37.9%). Initial imaging showed a TICI-AMI score of 0, 1 or 2a in 87.9% (*n* = 51) of patients. Post-therapeutic TICI-AMI scores improved significantly with 87.9% of patients grade 2b and better. Median lactate levels reduced from 2.7 (IQR 2.0–3.7) mg/dL (1–18) to 1.45 (IQR 0.99–1.90). Intestinal ischemia was documented in 79.1% of cases with resection of the infarcted intestinal loops. In total, 22/58 (37.9%) patients died during the first 30 days after intervention and surgery. According to CIRSE criteria, we did not observe any SAE scores of grade 2 or higher. Conclusions: AMI is a serious disease with high lethality within the first 30 days despite optimal treatment. However, interventional revascularization before surgery with resection of the infarcted bowel can save two out of three of critically ill patients.

## 1. Introduction

Mesenteric ischemia is most often defined as a complex of symptoms resulting from acute or chronic occlusion of the mesenteric vessels that supply the intestines. Occlusion initially leads to cellular damage, tissue death due to ischemia and later to secondary inflammatory changes [1]. In untreated cases, mesenteric ischemia leads to life-threatening intestinal necrosis. Although the incidence of mesenteric ischemia is relatively low (0.09–0.2% of all acute surgical hospital admissions), it should always be excluded as a differential diagnosis because mortality is reported in the literature to be as high as 50–80% [2,3,4,5]. However, early diagnosis of mesenteric ischemia can significantly reduce mortality. Acute mesenteric ischemia (AMI) can have a variety of causes: it may be non-occlusive (NOMI) or occlusive and caused by either arterial embolism (50%), arterial thrombosis (15–25%) or mesenteric venous thrombosis (5–15%) [6,7].

A mesenteric embolus may originate from the left atrium in cardiac arrhythmias or in global heart failure with a poor ejection fraction. Less frequently, such emboli originate from an arteriosclerotic aorta. These emboli typically attach to the narrowest part of the vessel, making the superior mesenteric artery (SMA) a predestined site in addition to its shallow angle of origin from the aorta and its relatively large lumen [8]. In particular, the area 3–10 cm downstream of the SMA is particularly vulnerable to occlusions (>20% of emboli), which supplies the main portion of the ileum.

Mesenteric arterial thrombosis is almost always associated with pre-existing chronic atherosclerosis. The vast majority of these patients have a history of symptomatic chronic mesenteric ischemia, such as postprandial pain and weight loss [8]. Because mesenteric thrombosis of the SMA most commonly has an underlying calcified plaque, the truncus is usually also involved [9]. However, SMA thrombosis may also occur in the setting of vasculitis, dissection or aneurysm.

Once the diagnosis of acute mesenteric ischemia is made by contrast-enhanced computed tomography, therapy should be initiated immediately. This includes the immediate administration of fluids and broad-spectrum antibiotics, endovascular revascularization and subsequent diagnostic laparoscopy, especially in patients with signs of peritonism [10]. In selected cases, surgical embolectomy may also be the procedure of choice.

To date, limited data is available for this therapeutic regimen. Hence, the aim of this study was to evaluate patient outcomes after interventional revascularization of the SMA, considering the degree and time of ischemia, as well as the technical success of revascularization.

## 2. Material and Methods

**Figure 1:**
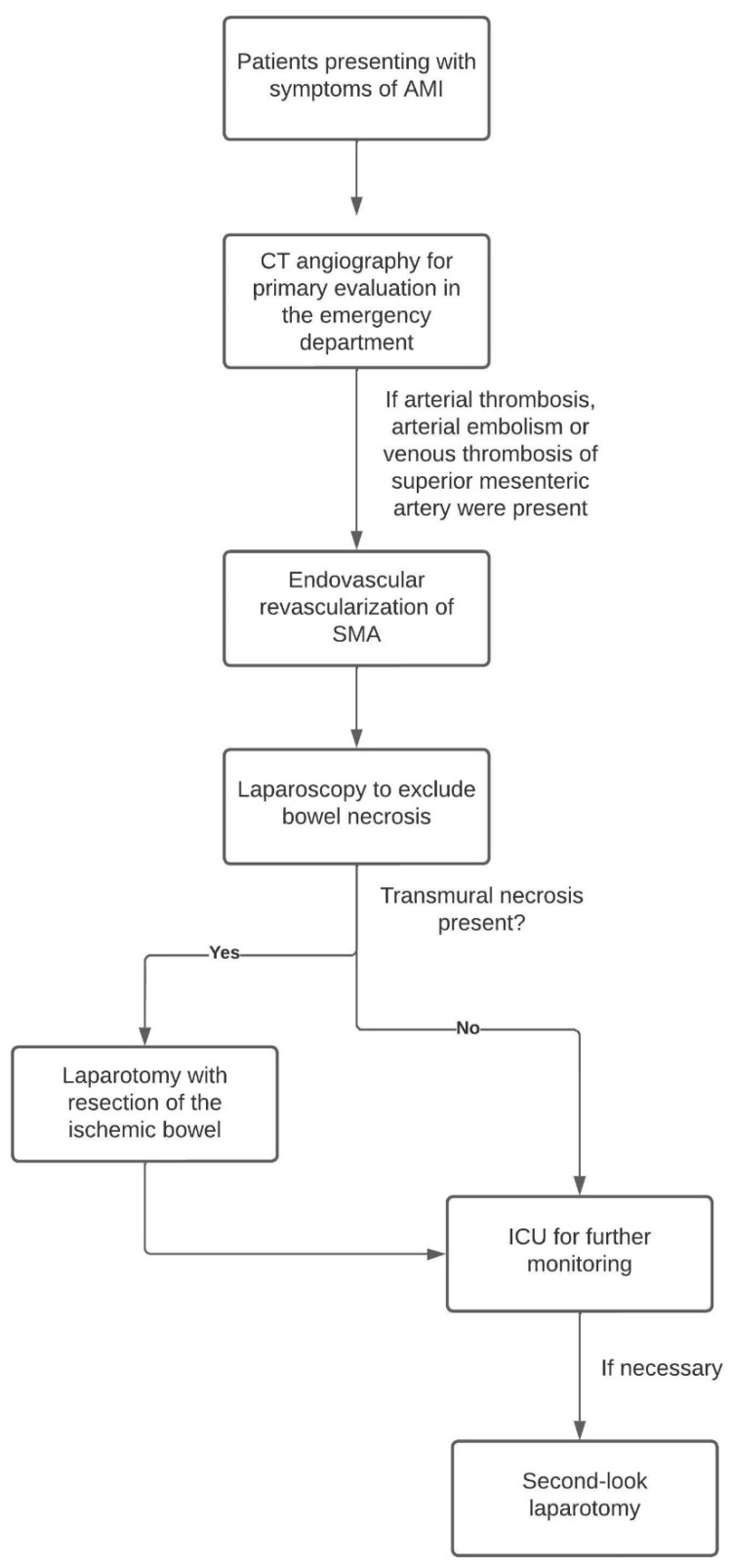
In-house procedure of treatment in patients presenting with AMI.

This study was conducted retrospectively at a single center and was IRB approved. Electronic medical records from our primary medical centre were screened in order to identify patients who presented with the signs and symptoms of AMI due to occlusion of the SMA between August 2016 and August 2021. We included consecutive patients with either arterial thrombosis, arterial embolism or venous thrombosis. We excluded patients with AMI with (A) non-occlusive mesenteric ischemia, (B) incomplete clinical data, (C) insufficient imaging or (D) missing follow-up laparoscopy (see flow chart Figure 1). Other data collected from the electronic medical records were: age at intervention, sex, weight, height, access location and tool, type of thrombectomy or embolectomy, additional procedures such as percutaneous transluminal angioplasty (PTA) or stents, reports of previous abdominal surgery, lab work including lactate and partial thromboplastin time (PTT), medication (e.g., amount of intraprocedural heparin or post-procedure anticoagulation) and pre-existing medical conditions, such as coronary artery disease, hypertension, diabetes and atrial fibrillation. Survial data were assessed after 30 days and 12 months.

All patients presenting to our clinic with clinical signs of AMI underwent contrast-enhanced CT for primary evaluation. All CT scans were performed on a second or third-generation CT scanner (Siemens Somatom Force or Somatom AS+, Siemens Healthineers, Erlangen, Germany). Iodinated contrast medium (Imeron 400, Bracco Imaging Deutschland GmbH) was administered at a dosage of 1.5 mL/kg and at a rate of 3.5 mL/s in every patient. Image acquisition was performed in native, arterial and portal venous contrast medium phases, respectively. Patients with imaging findings suggestive of acute mesenteric ischemia were immediately discussed by an interdisciplinary team of radiologists and abdominal surgeons and were included in the in-house treatment scheme of AMI.

### 2.1. Endovascular Procedure

The patients were immediately transferred to the interventional radiology angiography suite. All patients were treated either under general anesthesia or sedation. After sterile draping, arterial access was made via the left brachial artery or the right common femoral artery. The access site was chosen at the judgment of the executing interventionalist, considering the steepness of the SMA origin. Accordingly, the access sheaths were either a wire-reinforced 6 French 60 cm sheath (Terumo) from the arm or a 6F 45 cm renal double curved (Terumo) configured sheath from the groin. The occluded vessels were probed via 4F angiography catheters in a multipurpose configuration cranially or in C2 or S1 form caudally (Cook). Angiograms were acquired before recanalization. A 0.018-inch wire (Command 18, Abbott) was used for the initial crossing of the occlusion. Depending on the aetiology of the occlusion, an interventional treatment regime was set. Thrombectomy was performed with hydrodynamic thrombectomy, including power pulse spray lysis with 10 mg of alteplase (AngioJet *n* = 21), rotational thrombectomy (Rotarex 6F *n* = 2) or aspiration thrombectomy with a 6F guide catheter (VistaBrite 6F *n* = 28). Additional short-term lysis with a 10 mg alteplase bolus was given in *n* = 36 cases. Stenting or PTA of the underlying stenoses was performed as necessary. The interventional success of recanalization was finally confirmed by angiography. The transbrachially inserted sheaths were removed after successful PTA. The transfemoral accesses sheaths were often changed to short 6F sheaths and remained in place until after surgery. The resected bowel parts were documented and included in the further evaluation of this study.

### 2.2. Surgery

All patients were taken to the operating room following intervention and underwent diagnostic laparoscopy. In cases with ischemic bowel loops, resection adapted to the extent of irreversible ischemia was performed. Patients with bowel loops with possible reversible ischemic damage underwent second-look laparoscopy after 24 h for definitive care.

### 2.3. Anticoagulation

In cases with remaining embolic occlusions, patients received therapeutic full heparinization with a target PTT of 50 to 70 s. After final surgical treatment, the patients were loaded with aspirin (500 mg) and clopidogrel (300 mg) orally. All patients were recommended dual antiplatelet therapy with 100 mg of aspirin and 75 mg of clopidogrel daily for four weeks. This was followed by single platelet inhibition with aspirin (100 mg). All patients with a cardiogenic cause for an embolic event were subsequently treated with an appropriate plasmatic anticoagulant.

### 2.4. Outcome Assessment

The extent of AMI and the degree of technical success was graded according to a modified TICI-AMI (Thrombolysis in Cerebral Infarction scale) classification (0: no perfusion; 1: minimal; 2a: < 50% filling; 2b: > 50%; 2c: near complete or slow; 3: complete) [11]. Other important parameters evaluated were the time from symptom onset to angiography and the time from angiography to surgical evaluation of the necrotic bowel loops. Furthermore, a correlation between the outcome and multiple parameters, including the TICI score, were established.

### 2.5. Statistical Analysis

Statistical evaluation was performed using SPSS Statistics 27 (IBM, Armonk, NY, USA) and GraphPad (GraphPad Prism version 9.0.0 for Windows, GraphPad Software, San Diego, CA, USA, www.graphpad.com accessed on 5 November 2008). In the descriptive statistics, continuous values fulfilling a normal distribution were reported as mean values including standard deviations. Ordinal data were reported as medians with the 10th to 90th percentiles in parentheses. The data were tested for normal distribution using the Shapiro–Wilk test. *p*-values of α < 0.05 were considered statistically significant.

## 3. Results

A total of 58 patients (55% females) (median age 73.5 years [range: 43 to 96 years], 59% female, median BMI 26.2 kg/m^2^ [range: 16.0 to 39.2 kg/m^2^]) were included in this study.

The patients’ characteristics are summarized in Table 1.

Of the 58 patients enrolled, 100% had acute occlusion with acute symptoms. The predominant access site was brachial in 30 patients (51.7%) and femoral in 28 patients (48.3%), and the maximum sheath size was 6F in the vast majority of patients (87.9%). Other sheath sizes were 4F (*n* = 1, 1.7%), 5F (*n* = 3, 5.2%) and 7F (*n* = 2, 3.4%). The amount of heparin administered varied between 2500 units (3.4%), 5000 units (60.3%), 7500 units (5.2%) and 10,000 units (1.7%). A total of 17 patients (29.3%) received heparin via a perfusor during the intervention. Abdominal 3-phase CT angiography was conducted in 100% of cases. The extent of bowel ischemia was classified in the CT scan; the vast majority of patients had <50% initial (pre-interventional) filling of the mesenteric branches (89.6%). Pre-existing AMS stenosis was present in 74.1% of patients and a larger proportion of patients had embolic occlusion (51.7%), followed by mesenteric (arterial) thrombosis (37.9%). Mesenteric occlusion was often treated by a combined procedure of different recanalization methods, with aspiration thrombectomy in 48.3% of patients, systemic lysis in 55.2% of patients and hydrodynamic thrombectomy in 26.2% of patients. The mean time between the onset of symptoms to intervention was 222 min (Table 2). The TICI-AMI score before recanalization confirmed the CT results, with a large proportion of patients with <50% initially contrasted mesenteric vessels. After the intervention, the TICI AMI improved in total (Table 3).

In laparoscopy following the intervention, intestinal ischemia was present in 55.2% of patients; a mean of 110 cm of ileum was resected (Table 4). Three patients even received a complete colectomy in combination with partial ileum resection.

**Figure 2:**
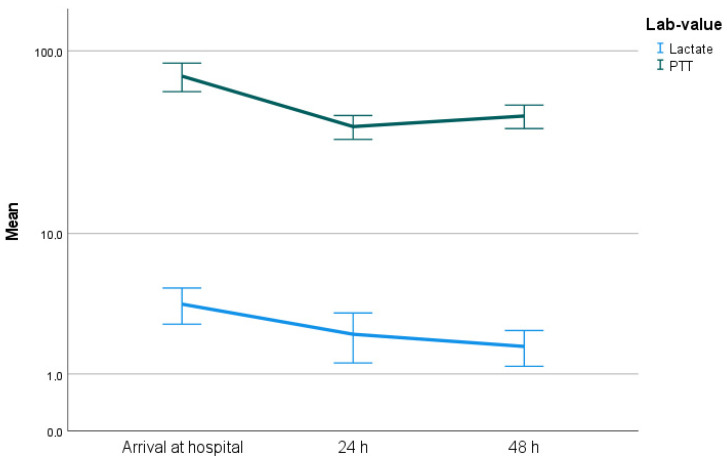
Significant decrease in serum lactate levels (*p* < 0.001) and in serum PTT (*p* < 0.001).

After intervention, serum lactate level dropped statistically significantly from a mean value of 4.26 mmol/L initially to 1.8 mmol/L after 24 h (*p* < 0.001) (Figure 2). 

**Figure 3:**
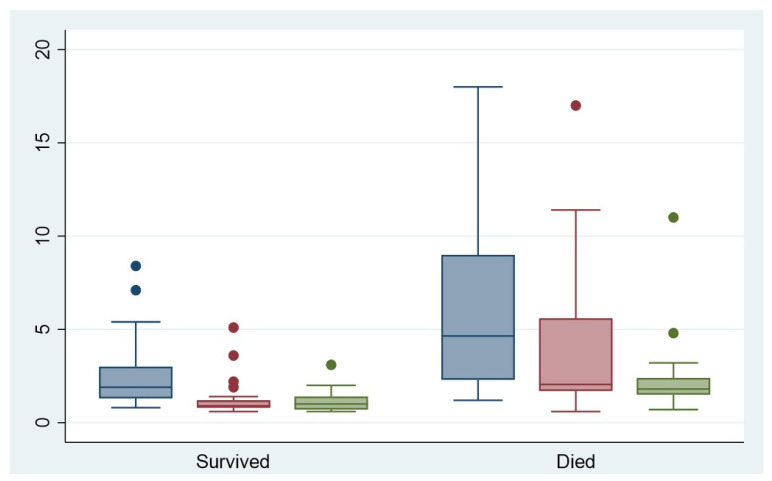
Serum lactate levels on arrival at hospital (blue), after 12 h (red) and after 24 h (green).

**Figure 4:**
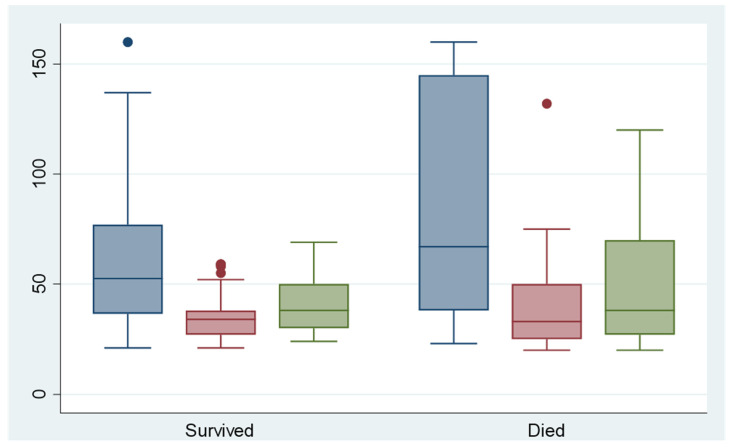
Serum PTT levels during angiography (blue), 12 h after angiography (red) and 24 h after angiography (green).

There were statistically significant differences in the laboratory values of lactate and PTT in relation to the arrival value at the hospital or at the onset of symptoms, and also in relation to the course of laboratory values during the first 24 h (Figure 3 and Figure 4). The patients who died within the first 12 months after symptom onset had significantly higher lactate and PTT initially and after 12 h (both *p* < 0.05).

**Figure 5:**
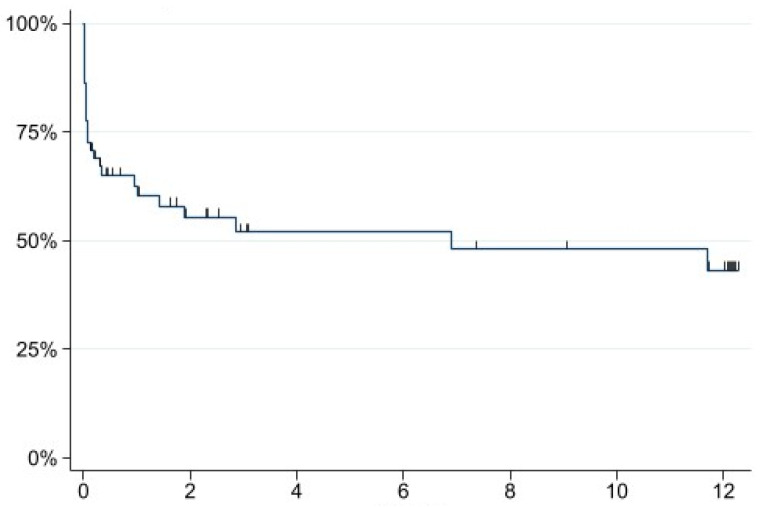
Kaplan–Meier mortality curve; *x*-axis: months; *y*-axis: probability of survival.

Kaplan–Meier estimates showed a 50% mortality within 6.9 months, whereas 25% mortality was already achieved at 0.67 months (Figure 5).

**Figure 6:**
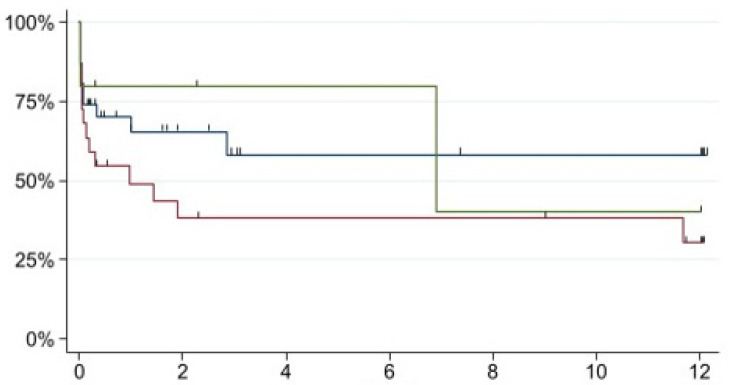
Kaplan–Meier estimate of the aetiology of mesenteric ischemia and patient death. The green line represents dissection, the blue line represents embolus and the red line represents thrombosis. Chi^2^ = 12.25, *p* = 0.006; *x*-axis: months; *y*-axis: probability of survival.

In our cohort, we did not find statistically significant correlations between the aetiology of mesenteric ischemia and patient death using the log-rank test (Chi^2^ = 2.52, *p* = 0.28). However, we found a statistically significant correlation with respect to the extent of intestinal ischemia (see Figure 6 Chi = 12.25, *p* = 0.006). There were other factors that did not have a statistically significant effect on death (see Table 5).

There was no statistically significant association between the aetiology of ischemia and overall survival during the first 12 months (*p* = 0.28, see Figure 6). Although all three aetiologies showed high initial mortality, mesenteric thrombosis showed a slightly increased mortality compared with embolism.

An interesting side-fact resulting from our data was that resection of the ileum clearly correlated with survival; patients who had a portion of ileum resected had a 2.3-fold increased risk of death (*p* = 0.02). Furthermore, time from symptom onset statistically significantly correlated with patient mortality (*p* = 0.043), whereas patients with a shorter time period had a significantly longer survival (Table 6).

## 4. Discussion

Acute mesenteric ischemia is a life-threatening condition that requires immediate treatment. Acute thrombosis of the ostia of the superior mesenteric artery has been described to be associated with the worst prognosis, since the affected patient population is mostly of advanced age with concomitant atherosclerosis and pre-existing stenosis of the ostium [4,12,13]. In this retrospective analysis, 74.1% of patients had a pre-existing stenosis of the ostium of the SMA. This, in the case of acute occlusion, usually involves a larger part of the bowel, in contrast to the purely arterial embolus, which might usually be located more distally in the vascular tree. Especially problematic in the context of mesenteric ischemia are elderly patients with atrial fibrillation who develop an embolic occlusion. In contrast to patients with chronic stenosis, these patients do not (yet) have any bypass circuits. There was no statistically significant correlation between death and aetiology of mesenteric ischemia (*p* = 0.28).

It is widely recognized that time is critical in the treatment of AMI. Therefore, the restoration of blood supply to the bowel is the first priority in these patients in order to avoid the occurrence of irreversible necrosis of the bowel wall. Nevertheless, the results of delayed diagnosis or treatment in predicting mortality after AMI are controversial in the literature [14,15], and current data suggest that ischemic changes are reversible in the first 6 h, although this is in contrast to ischemic stroke. Furthermore, current guidelines do not provide precise information on the correct timing for revascularization. In our cohort, a mean time between the onset of symptoms and revascularization of 222 min (=less than 4 h) could be achieved, which lead to improved survival compared to the existing literature. In our cohort, there was a statistically significant difference in terms of patient survival and the time from symptom onset to intervention.

Regarding the poor prognosis of patients with AMI and the fact that time plays a crucial role in the prevention of intestinal necrosis and survival [16], it would be useful to identify factors that can predict the outcome of patients after revascularization in order to improve treatment.

Several studies have suggested that elevated serum lactate levels, which result from anaerobic glycolysis, are associated with irreversible transmural necrosis [17,18] and a worse outcome [19,20]. Our results indicate a clear distinction between the laboratory parameters of lactate and PTT with respect to the subgroups “dead within 12 months” and “alive after 12 months” (both *p* < 0.05). This distinction is associated with the extent of mesenteric ischemia, as patients with extensive ischemia are also more likely to have a shorter survival time.

These factors could be indicators for poor outcomes and alternative treatment options as open surgery or hybrid retrograde open mesenteric stenting could be considered in these patients, whereas retrograde stenting requires dedicated preparation, but has shown promising results [21].

Another important predictor of the outcome for patients with mesenteric ischemia is the presence of pre-existing co-morbidities. In our cohort, 50 out of 58 patients had various pre-existing co-morbidities, of which 25 had CHD, 33 had hypertension, 11 had diabetes, 21 had cardiac arrhythmias, 15 suffered from chronic peripheral arterial occlusive disease and 9 patients were undergoing dialysis. None of these proved to be reliable in predicting the patients’ outcome, which is consistent with the literature [12].

The limitations of this study include its retrospective nature and limited cohort size, as it was performed at a single centre and only in patients with clinically significant occlusion of the SMA, whereas patients with occlusion of the celiac trunk were excluded. In addition, all the patients were treated under emergency conditions by different interventionalists and there was no standardized follow-up.

In conclusion, the 30-day mortality rate in patients with AMI (independent of aetiology) remains high despite emergency endovascular revascularization/stenting of the SMA. In the absence of statistically reliable prognostic factors for clinical outcomes in this patient population that could potentially be used to guide patient management toward alternative treatments, we recommend immediate endovascular revascularization followed by surgical screening for ischemic bowel parts in all patients.

## Figures and Tables

**Table 1:** Patient characteristics at the time of mesenteric ischemia. All the values are given as median ± standard deviation. BMI: body mass index.

Age (mean years ± SD)	71.8 ± 14.4
BMI (mean kg/m^2^ ± SD)	25.8 ± 5.3
Sex (m/f)	32 females (55.2%) 26 males (44.8%)
Coronary heart disease present?	*n* = 25 (43.1%)
Hypertension present?	*n* = 33 (56.9%)
Diabetes present?	*n* = 11 (19.0%)
Atrial fibrillation present?	*n* = 21 (36.2%)
Chronic peripheral arterial occlusive disease present?	*n* = 15 (25.9%)
Dialysis present?	*n* = 9 (15.5%)

**Table 2:** Details of Intervention.

Access Vessel	
Brachial	*n* = 31 (53.4%)
Femoral	*n* = 27 (46.6%)
Sheath size	
4 French	*n* = 1 (1.7%)
5 French	*n* = 3 (5.2%)
6 French	*n* = 52 (89.6%)
7 French	*n* = 2 (3.4%)
Complications	
Access site	*n* = 0 (0%)
Mesenteric arteries	*n* = 0 (0%)
Administered Heparin during procedure	
2500 i.u.	*n* = 2 (3.4%)
5000 i.u.	*n* = 25 (60.3%)
7500 i.u.	*n* = 3 (5.2%)
10,000 i.u.	*n* = 1 (1.7%)
Continuous therapeutic heparinization	*n* = 17 (29.3%)
Pre-interventional imaging	
Abdominal CT Angiography	*n* = 58 (100%)
Extent of bowel ischemia on CT	
0: no peripheral perfusion	*n* = 22 (37.9%)
1: minimal	*n* = 4 (6.9%)
2a: <50% filling	*n* = 26 (44.8%)
2b: >50% filling	*n* = 4 (6.9%)
2c: near complete	*n* = 0 (0%)
3: complete	*n* = 0 (0%)
Pre-existing AMS stenosis	
Yes	*n* = 43 (74.1%)
No	*n* = 15 (25.9%)
Type of occlusion	
Embolus	*n* = 30 (51.7%)
Thrombosis	*n* = 22 (37.9%)
Dissection	*n* = 4 (6.9%)
Vasculitis	*n* = 2 (3.4%)
Time onset of symptoms to intervention (mean min ± SD)	
	222 min ± 166 min
Method(s) of recanalization	
Primary stent	*n* = 16 (27.6%)
Hydrodynamic thrombectomy	*n* = 21 (36.2%)
Rotational thrombectomy	*n* = 2 (3.4%)
Aspiration thrombectomy	*n* = 28 (48.3%)
Lysis	*n* = 32 (55.2%)
Additional	
Balloon angioplasty	*n* = 31 (53.4%)
Stent	*n* = 14 (24.1%)

**Table 3:** Change in TICI AMI before and after recanalization.

TICI AMI before recanalization 0: no peripheral perfusion 1: minimal 2a: < 50% filling 2b: > 50% filling 2c: near complete or slow 3: complete	*n* = 2 (3.4%) *n* = 24 (41.4%) *n* = 25 (43.1%) *n* = 6 (10.3%) *n* = 1 (1.7%) *n* = 0 (0%)
TICI AMI after recanalization 0: no peripheral perfusion 1: minimal 2a: < 50% filling 2b: > 50% filling 2c: near complete of slow 3: complete	*n* = 0 (0%) *n* = 4 (6.9%) *n* = 3 (5.2%) *n* = 17 (29.3%) *n* = 19 (32.8%) *n* = 15 (25.9%)

**Table 4:** Results of surgical follow up after intervention.

Time interval between end of intervention and start of laparoscopy (mean min ± SD)	71 min ± 42 min
Bowel ischemia in laparoscopy Yes No	*n* = 32 (55.2%) *n* = 26 (44.8%)
Extend of bowel resection Ileum Right hemicolectomy Total colectomy	Mean about 110 cm *n* = 5 *n* = 3

**Table 5:** Log-rank test between several clinical parameters and death. All parameters were obtained as part of the treatment regimen for mesenteric ischemia.

Death	Chi^2^	*p*-Value
Aetiology of mesenteric ischemia	2.52	0.28
Extent of intestinal ischemia	12.25	0.006
Resection of colon	1.37	0.24
Patient history of coronary heart disease	0.76	0.09
Patient history of hypertension	0.51	0.43
Patient history of diabetes	0.38	0.54
Patient history of atrial fibrillation	0.49	0.48
Patient history of chronic peripheral occlusive disease	0.6	0.44
Patient history of dialysis	0.55	0.46

**Table 6:** Multivariate analysis of “time from symptom onset”, “resection of ileum” and “survival” of patients.

Correlation	Time from Symptom Onset	Survival	Resection of Ileum
Time from symptom onset	x	*r* = −0.220, *p* = 0.043	*r* = −0.047, *p* = 0.73
Survival	*r* = −0.220, *p* = 0.043	x	*r* = 0.316, *p* = 0.01
Resection of ileum	*r* = −0.047, *p* = 0.73	*r* = 0.316, *p* = 0.01	x
